# Application of Small Specimen Test Technique to Evaluate Creep Behavior of Austenitic Stainless Steel

**DOI:** 10.3390/ma12162541

**Published:** 2019-08-09

**Authors:** Bintao Yu, Wentuo Han, Zhenfeng Tong, Diancheng Geng, Chenlong Wang, Yingchao Zhao, Wen Yang

**Affiliations:** 1 China Institute of Atomic Energy, Beijing 102413, China; 2 School of Materials Science and Engineering, University of Science and Technology Beijing, Beijing 100083, China

**Keywords:** creep, small specimen test techniques, fast reactor, austenitic steel, precipitation

## Abstract

Small specimen test techniques (SSTT) are highly demanded in the nuclear field. In the present work, SSTT was applied to the creep tests of 15-15Ti austenitic steel. The creep behaviors of specimens with miniaturized and standard sizes were contrastively studied. The feasibility of SSTT is verified after tests under more than 20 creep conditions. The results that were obtained by miniaturized specimens are relatively conservative and they can be securely applied. The stress exponent and apparent activation creep energy of 15-15Ti are calculated as 7.7 and 428 kJ/mol, respectively. The creep microstructures are characterized by the evolution of dislocations, deformation twins, and precipitates.

## 1. Introduction

At present, the 600 MW demonstration fast reactor is under construction in China, and the domestic production of the key components is also on the agenda. As an essential part of the fast reactor, fuel cladding protects the fuel pellets from the corrosion of coolant, prevents the leakage of fission products, and provides sufficient strength and stiffness for fuel. The cladding suffers from the impact of fission gas, corrosion and erosion of coolant, intense neutron irradiation, thermal stress, thermal cycle and fuel swelling, etc., due to the severe working conditions [1]. For the safe operation of the reactor, the cladding should meet the integrity requirements and keep sufficient mechanical properties at elevated temperatures.

Internationally, austenitic stainless steel is generally chosen as the fast cladding material. China has selected 15-15Ti austenitic stainless steel as a promising candidate cladding material for the demonstration fast reactor, and devoted significant effort to the domestic production. The creep properties of 15-15Ti at high temperature before and after irradiation must be confirmed prior to the future application for safe operation of the fast reactor.

It is infeasible to irradiate sufficient number of the conventional rod-shaped creep specimens with large sizes due to the very limited space of the irradiation channel in the reactor. Additionally, the large mass of specimens can strongly produce the irradiation-induced radioactivity that are harmful to the experiment safety and can seriously impede the subsequent mechanical tests. The interest of applying small specimen tests is also supposedly, because the limited amount of material at disposal for being tested along the lifetime of the reactor.

Work to date has developed an array of techniques to extract mechanical properties—such as tensile data, fracture toughness, impact properties, fatigue, and fatigue crack growth—from relatively small specimens [2,3,4,5]. In contrast to the well-developed small specimen test technique (SSTT) of the above properties, the SSTT data of creep evaluation are relatively limited. Therefore, it is urgent to establish the small specimen test technique (SSTT), in particular that to evaluate the creep property of the cladding material.

The attempts for SSTT of creep evaluation have been done by sub-sized uniaxial creep test (SUCT), impression creep test (ICT), small punch creep test (SPT), small ring creep test (SRT), and small two-bar specimen creep test (TBT) [6,7,8]. In these methods, only the SUCT, SPT, and TBT methods are capable of producing comprehensive creep data, such as creep strain rate and rupture life. However, the SPT and TBT strictly require tests to perform in non-oxidation environment that highly limited the practical application [6]. In sharp contrast, the SUCT have been widely applied in severe environments, such as flowing lead-bismuth eutectic (LBE) [9] and stagnant Pb with various amounts of dissolved oxygen [10].

In the references of SUCT, the creep specimens are mainly with cylindrical shapes, and the minimum end diameter is about 5 mm in the M5 type specimen [11,12,13,14]. The M5 specimen shows a good agreement with conventional larger specimens. When considering the limited capacity of irradiation chambers and the large amount samples required for creep tests, the size of M5 is still too large. Using miniaturized sheet specimens could be an alternative way. The sub-sized sheet specimens, such as SS-1, SS-2, SS-3, and SS-J3 have been successfully applied in the SSTT of tensile properties [15,16,17]. However, no systematic study has been done for obtaining the creep properties by miniaturized sheet specimens.

## 2. Experimental Details

**Figure 1:**
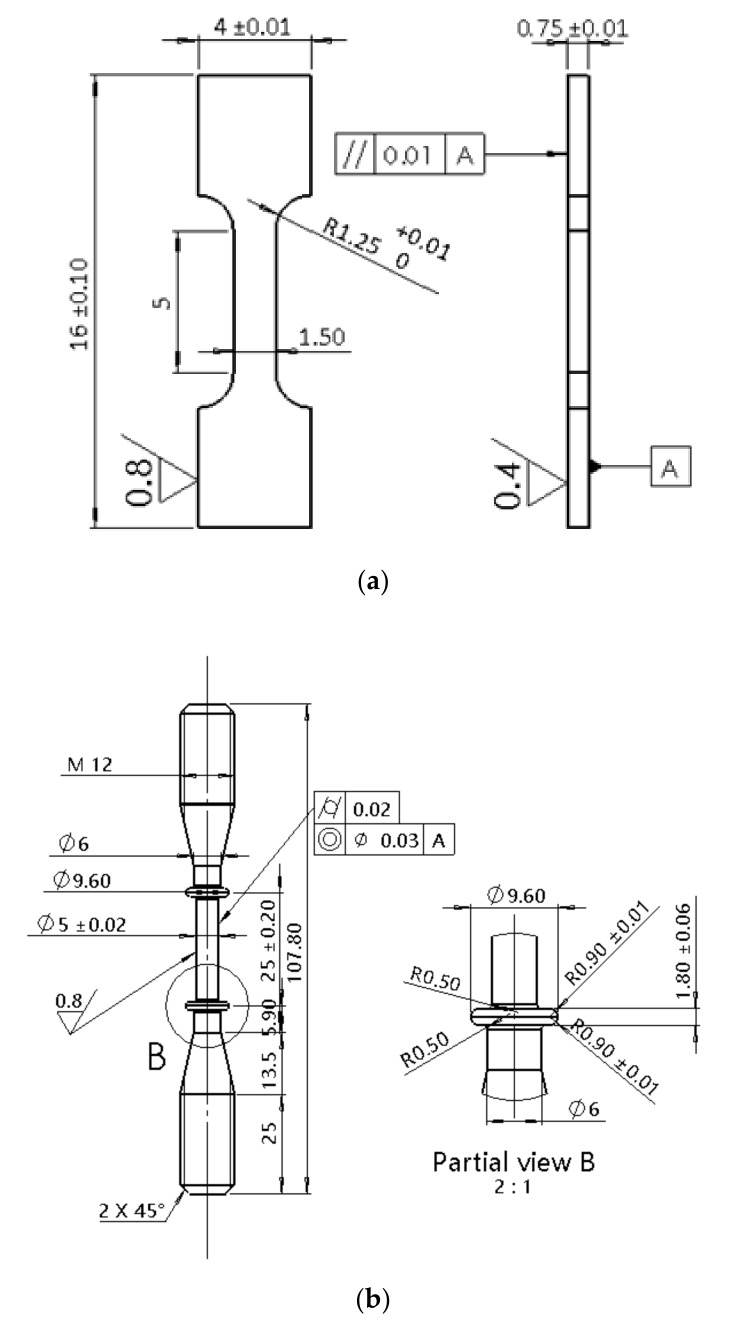
Specimen dimensions: (**a**) miniaturized sheet specimen with a length of 16 mm; (**b**) standard creep specimen with a rod shape and a 108 mm length.

The material used in this study was a 15-15Ti austenitic stainless steel with the composition that is detailed in Table 1. The as-received material has undergone a 20% cold work and had an average grain size of 11.3 µm. For investigating the effects of specimen size on the creep behaviors, standard rod specimens with a length of 170.8 mm were fabricated according to ISO 204 [18] and set as a reference, while the sub-sized sheet specimens were dimensioned in 16 mm length, 1.5 mm width, and 0.75 mm thickness. The standard and the sub-sized specimens were both cut from the steel bars, which had a diameter of 18 mm. The lengthwise direction of the creep specimens was paralleled to the long axis of the steel bar. Figure 1 presents the detailed specimen shape and size.

**Figure 2:**
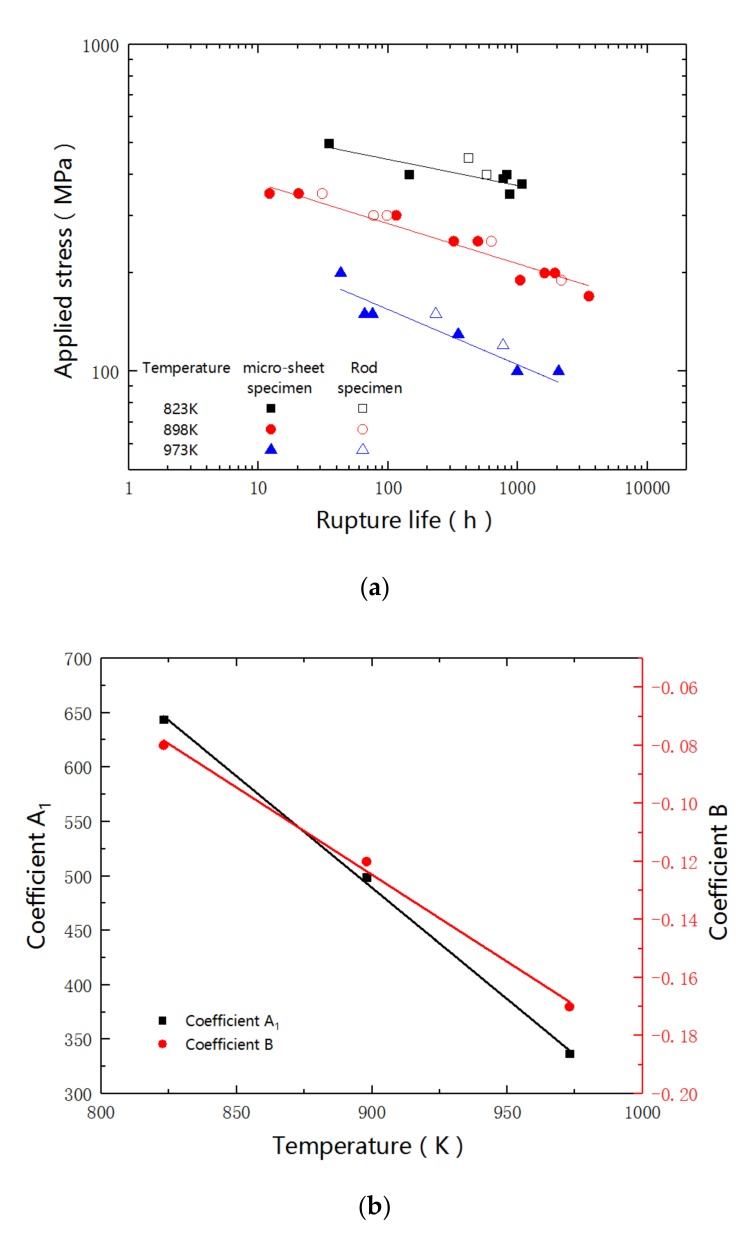
Creep test results: (**a**) data summary and comparison between the sub-sized sheet specimens with standard specimens; and, (**b**) dependence of coefficients for estimating the rupture life on temperatures.

The creep behaviors were tested by lever creep testing machines (MTS-GWT1104) (produced by MTS Systems (China) Co., Ltd, Shenzhen, China), with stresses ranging from 100 MPa to 500 MPa, while the test temperatures were set as 823, 898, and 973 K. With the different applied stresses and test temperatures, more than 20 creep conditions were subjected to 15-15Ti steels, as detailed in Figure 2a. The specimen elongation during the tests was measured by monitoring the grip movement. A grating ruler was used for measuring and recording elongations. Totally, more than 20 creep conditions were subjected to the 15-15Ti. Transmission electron microscopy (TEM) was applied to analyze the microstructures after creep tests. High-angle annular dark-field (HAADF) and weak-beam dark-field (WBDF) microscopy techniques were used to investigate the precipitate evolution.

## 3. Results and Discussion

After being tested under more than 20 creep conditions, the obtained results are summarized in Figure 2. The miniaturized specimens show similar tendencies with the standard ones that, at each testing temperatures, increasing the stress can shorten the creep life. For instance, at 898 K, the standard specimen and the sub-sized specimen under 250 MPa have rupture times of 626 h and 490 h, respectively, while the rupture times of both specimens that were subjected to 300 MPa decrease to about 100 h. Remarkably, the stress and creep life of both specimens exhibit linear relation in the logarithmic coordinates. The relation can be described, as follows [19]:

σ = A_1_ × t_r_^B^ (1)

where σ is the applied stress, t_r_ is the creep life, and A_1_ and B are the coefficients.

By fitting the curve, the coefficients of A_1_ and B at each testing temperature can be obtained, as listed in Table 2. The coefficients of A_1_ and B continuously decrease with the increase of temperature. By plotting A_1_ and B with the testing temperature, linearity relations are exhibited in Figure 2b. The relation can be formulated, as follows:

A_1_ = −2.05 × T + 2331 (2)

B = −0.0006 × T + 0.42(3)

The Equation (1) can be further detailed as:

σ = (−2.05 × T + 2331) × t_r_^−0.0006 × T+0.42^ (4)

The Equation (4) not only reveals the relation between the stress and the creep life, but it also provides an easy way to estimate the creep life for 15-15Ti by the given stress and testing temperature.

Although lgσ and lgt_r_ preferably meet a linear relation in the presented data, it has been reported that this method is not faithful to be applied to long-term extrapolating in a wide stress range [18,20]. A great number of experimental data [19,20,21,22] indicated that the steady state creep rate plays an important role in the creep life.

**Figure 3:**
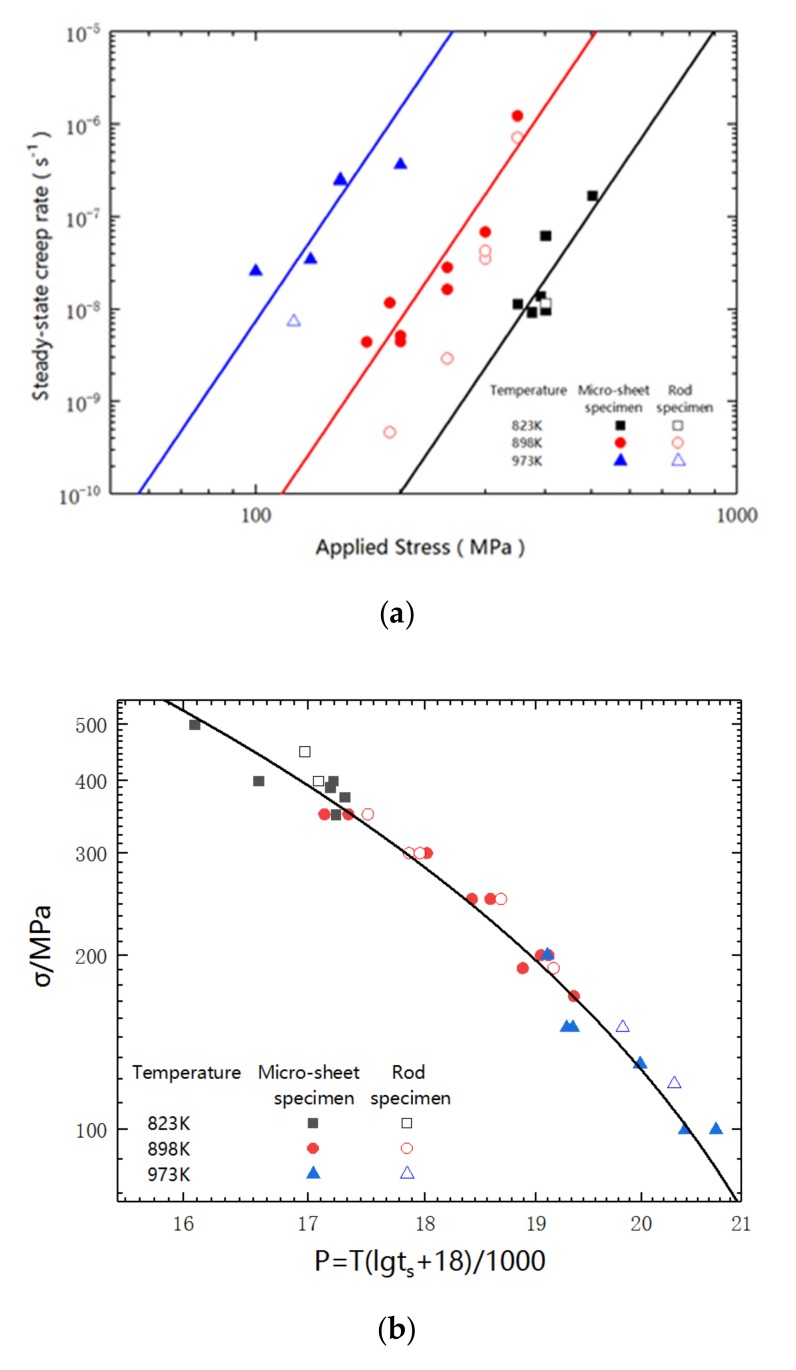

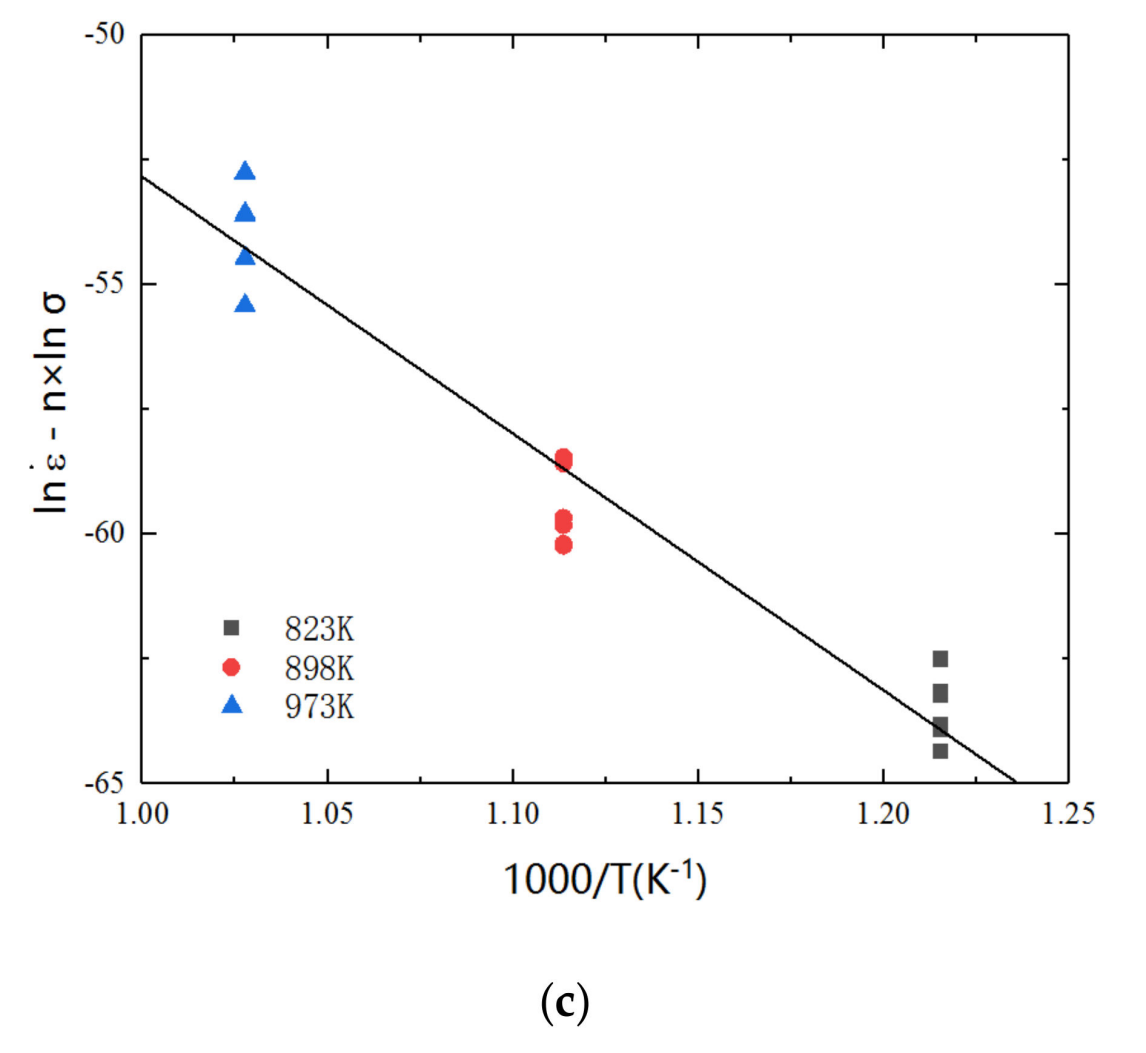
Creep data processing: (**a**) summary of stress-steady state creep rate versus applied stress; (**b**) applied stress versus creep life in Larson-Miller expression; and, (**c**) data fitting for the calculation of apparent activation energy.

The steady-state creep rate and the stress are summarized and diagrammed in Figure 3a. Linear relation is obviously exhibited. The miniaturized specimens have very similar slopes at three different testing temperatures. Noticeably, with the same subjected stress, the miniaturized specimen owns a bigger creep rate than that of the standard rod-shaped specimen. The results, as obtained from both specimen types, should be the compensation between the length effect (25 mm free length for the standard specimens and 5 mm for the sub-sized specimens) and the cross section counter effect (19.6 mm^2^ in sectional area for the standard specimens and 1.13 mm^2^ for the sub-sized specimens). Besides the size of length and cross section, the different specific surface areas could also affect the stress distribution. The angular shape of the sheet specimen is generally considered to be more vulnerable to the stress concentration. Therefore, with the same subjected stress, the creep rate of the miniaturized specimen is higher than that of the stranded rod specimen. The miniaturized specimen has a relatively shorter rupture life than that of the standard specimen due to the higher creep rate, as shown in Figure 2a.

To describe the relation between creep life and the steady state creep rate, the following equation of Monkman–Grant [19,21,22] relation can be used:

(5) $$t_{r}\;\times \;\dot{\varepsilon }=C$$

where C is the constant related to the material, while the steady state creep rate, $\dot{\varepsilon }$, can be expressed by the Arrhenius equation [19,23]:

(6) $$\dot{\varepsilon }=A_{2}\sigma^{n}\exp\left(-\frac{Q_{c}}{\mathrm{RT}}\right)$$

In Formula (6), A_2_ is constant, n is stress is creep stress exponent, Q_c_ is apparent activation energy and R is gas constant.

According to (6), the Monkman–Grant relation can be transformed:

(7) $$\mathrm{lg}t_{r}=\frac{P_{1}}{T}-P_{2}$$

Suppose that P_1_ is a function of stress, and then Formula (7) fits Larson–Miller equation [24], as follows:

(8) $$P\left(\sigma \right)=T\left(C+{\mathrm{lg}\;t}_{r}\right)$$

As substitute the presented creep data into Formula (8), C = 18 could be worked out and the equation could be transformed, as follows:

(9) $$P\left(\sigma \right)=2658\times \left(8.34-{\left(\mathrm{lg}\sigma -1.20\right)}^{2}\right)=T\left(18+\mathrm{lg}t_{r}\right)$$

According to the Larson–Miller expression, the creep data are plotted in Figure 3b. The rupture times of both the miniaturized and large specimens are in good agreement with the fitting curve.

Based on the above findings, the results of miniaturized and standard specimens show good agreement. In addition, in most cases, the creep life that was obtained by the sub-size specimen is shorter than that of the standard specimen. For instance, the creep lives of the standard specimen are 626 h and 31 h under 250 MPa and 350 MPa, respectively, while the results of the sub-sized specimen are 490 h (250 MPa) and 16 h (300 MPa). Therefore, the presented miniaturized specimen can be used to evaluate the creep behaviors of 15-15Ti, and provide a relatively conservative estimate of the creep properties, which is conducive to being securely applied.

By further analyzing the data in Figure 3a, the creep stress exponent n of the miniaturized specimens is calculated as 7.7. The n value provides important information regarding the deformation mechanism. The literatures [19,25,26] report that a value of n = 1 happens in creep diffusion, n = 2 belongs to the grain boundary sliding controlled creep, and a value of n = 3–5 reveals the main creep mechanisms in pure metals and simple solid solution alloys are the dislocation creep. The n value is highly related to the microstructures. The n value can be drastically promoted in the precipitation hardened/dispersion strengthened materials. Miura et al. [27] found that the n value is 26 in an oxide dispersion strengthened (ODS) nickel-based superalloy MA754. Zakine et al. [28] reported that, in an ODS steel, the n values are in the range of 11~25. In addition, the authors found that the size of oxide particle can significantly affect the n value. With an average particle size of 80 nm, the n values are 11~13, while decreasing the particle size to 20 nm can enhance the n values to 17~25. The obtained n value of 7.7 suggests that the creep mechanism of 15-15Ti in the presented condition mainly belongs to the dislocation climb, while the precipitates in 15-15Ti are a benefit to the enhancement of the n value, as the precipitates can inhibit the dislocation motion.

Besides of the creep stress exponent n, the apparent activation energy is highly desired. In accordance with Formula (6), the following formula could be worked out:

(10) $$\ln\dot{\;\varepsilon }-n\;\ln\;\sigma ={\ln\;A}_{2}-\frac{Q_{c}}{R}\times \frac{1}{T}$$

In Formula (10), n is creep stress exponent, A_2_ is constant, Q_c_ is apparent activation energy, and R is the gas constant with a value of 8.314 J/(mol·K).

Based on the experimental data, the values of $\ln\;\dot{\varepsilon }-n\;\ln\;\sigma$ are calculated and plotted in accordance with the 1/T in Figure 3c. By fitting the data, the slope $\frac{Q_{c}}{R}$ can be calculated, and therefore the apparent activation energy, Q_c_, is decided as 428 kJ/mol. The obtained energy is obviously higher than the bulk diffusion activation energy (270 kJ/mol) and the boundary and pipe diffusion activation energy (159 kJ/mol) of γ-iron [23,29], but it is quite similar with that in other commercial alloys, such as 14Cr-15Ni-Ti (460 kJ/mol) [23], 316H (397 kJ/mol) [30], and 347SS (469 kJ/mol) [31]. The high level of the obtained activation energy is considered to be related with the precipitations in the commercial alloys.

**Figure 4:**
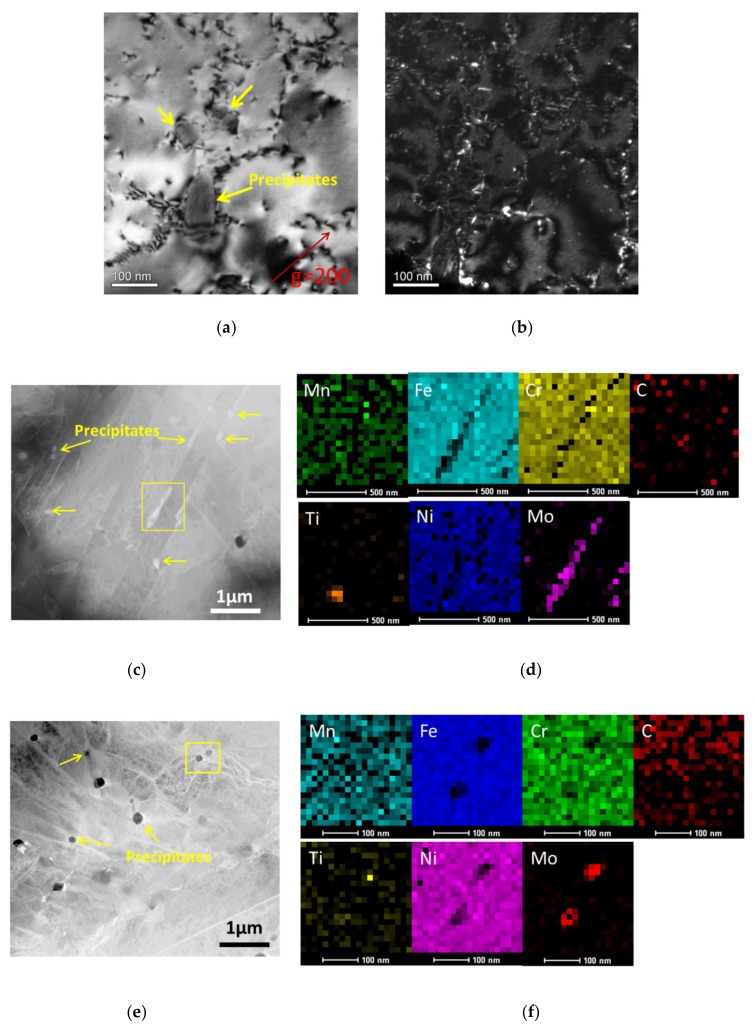
Precipitate observations: (**a**) transmission electron microscopy (TEM) bright field image and (**b**) weak-beam dark-field (WBDF) image after the creep test of 190 MPa; (**c**) high-angle annular dark-field (HAADF) image and (**d**) EDS images of the precipitate marked by square in the HAADF image after 190 MPa test; (**e**) HAADF image; and, (**f**) EDS images of the precipitate marked by square in the HAADF image after 300 MPa test.

The TEM observations of precipitates in the creep-tested 15-15Ti are representatively presented in Figure 4a,b. Dislocation pile-up is found to surround the precipitates that verifies the interactions between the precipitates and dislocations during creep. The precipitates are more discernible under HAADF, as shown in Figure 4c. The EDS results of the 190 MPa-ruptured specimen reveal that the precipitates are enriched in Mo, but depleted in Fe, Cr, and Ni. In addition, the Mo-enriched precipitates tendentiously appear on grain boundaries.

The Mo-enriched precipitates are also detected in the 300 MPa-ruptured specimen. However, the precipitates have a much smaller size, a spherical shape, and a finer dispersion, when comparing with that of the 190 MPa specimen. The differences in precipitates of 190 MPa and 300 MPa should relate with the creep test conditions. Although 190 MPa and 300 MPa are subjected at the same temperature of 898 K, the testing durations are different. With the applied stress of 300 MPa, the rupture happens at 115 h, while the testing time is 1045 h in the case of 190 MPa, as shown in Figure 2a. The overtime maintaining under thermal conditions can doubtlessly enhance the element diffusion, and therefore coarsen the precipitates in the 190 MPa-specimen. As the hindrance of dislocation motion strongly depends on the size, shape, and dispersion of the precipitates, the varied precipitations can affect the creep behaviors. As shown in Figure 2a and Figure 3b, the tested rupture life of 300 MPa and 190 MPa are higher and lower than the estimated creep life that was indicated by the fitting curves, respectively.

**Figure 5:**
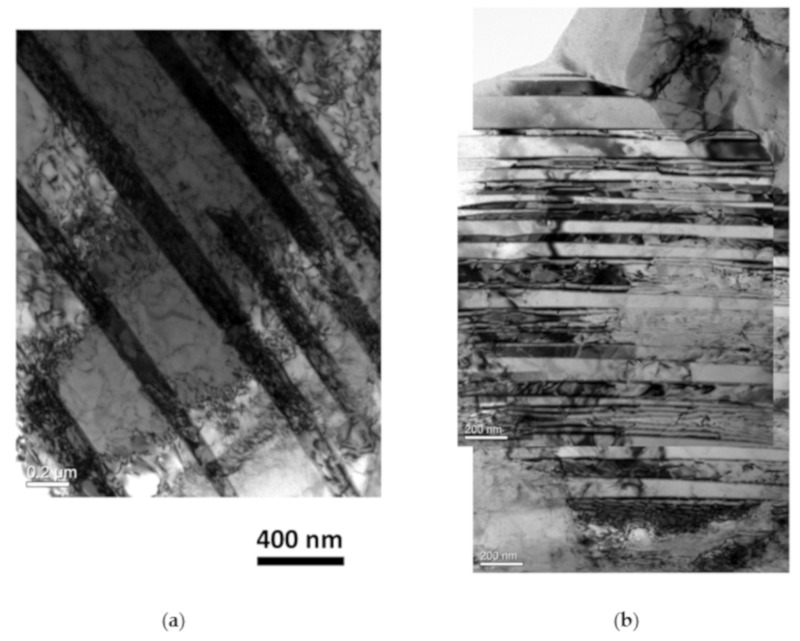
TEM observations of the deformation twins after creep tests of (**a**) 190 MPa and (**b**) 300 MPa.

Besides the varied precipitations, the deformation behaviors may also be affected by the value of applied stresses. The deformed microstructures at 898 K under 190 MPa and 300 MPa are representatively presented in Figure 5. Common things can be found in these conditions that deformation twins are widely generated during the creep tests. Additionally, more twins and drastic twinning deformation are detected under the high stress condition of 300 MPa. Numerous works have proved that twinning is an important deformation mechanism in the austenite deformation that can enhance the deformability, even in extreme conditions [32]. The favorable twinning during creep in 15-15Ti may also crucially contribute to the creep properties.

## 4. Conclusions

In summary, SSTT was applied to evaluate the creep properties of 15-15Ti austenitic steel. The miniaturized sheet specimen with a length of 16 mm shows a similar creep life with the standard rod specimen with a length of 108 mm. The creep data of the two type specimens agree well with the Larson–Miller fitting curve. The SSTT data is considered to be conservative, as in most cases the creep life provided by the miniaturized specimen is subtlety shorter than that of the standard specimen. The stress exponent and the apparent activation creep energy are calculated as 7.7 and 428 kJ/mol, respectively. The deformation mechanism mainly belongs to the dislocation creep, while the precipitate evolution and twinning deformation can also affect the creep behaviors.

Future work will be focused on applying SSTT to the creep tests in the irradiated 15-15Ti and characterizing the precipitate evolution during different creep stages.

## Figures and Tables

**Table 1:** Chemical composition of the austenitic stainless steel 15-15Ti (wt.%).

Element	Ni	Cr	Ti	Mo	Mn	Si	C	N	P	S	V	Cu	Al	Fe
Concentration	15.37	16.65	0.46	2.15	1.92	0.43	0.07	0.013	0.006	0.004	0.18	0.02	0.02	Balance

**Table 2:** Coefficient in the relationship of creep life and stress at different temperatures.

Temperature	A_1_	B
823 K	644	−0.08
898 K	499	−0.12
973 K	337	−0.17
